# Supplementary material for: Swedish Trotting Horse Trainers’ Perceptions of Animal Welfare Inspections from Public and Private Actors
Source: Animals (Basel). 2022 Jun 3;12(11):1441. doi: 10.3390/ani12111441 (PMC9179459; doi:10.3390/ani12111441)
Supplement: Supplementary file 1 [file animals-12-01441-s001.zip › animals-1693610-Questionnaire.pdf]

Compiled version of questionnaire translated from Swedish to English

A questionnaire on expectations and experiences related to animal welfare inspections in Sweden

There is an ongoing research project at the Swedish University of Agricultural Sciences (SLU) on how animal keepers and inspectors perceive different kind of animal welfare inspections. This questionnaire is addressed to you, as a trotting-horse trainer, which will get inspected both by the County Administrative Boards (CAB) and the Swedish Horse Trotting Association (STA).

The questionnaire is voluntary and no identifying information on you will be collected, i.e. we will not be able to see who has responded.

The aim with this project is to get a greater understanding on your and your colleagues' experience when having your stables and horses inspected. How do you perceive being inspected by two different actors, and is it possible to make the inspections more efficient? The project takes place in collaboration with STA and the Swedish Board of Agriculture (SBA), amongst others.

The questionnaire consists of four parts:
- information on you and your horse trotting business
- your experience on having inspections from different actors
- your expectations and experiences on animal welfare legislation and the official CAB inspections
- your expectations and experiences on the Trotter Health Standard and STAs inspections.

Part 1. Information on you and your horse trotting business

1. In which county (region) of Sweden do you have your main business?

2. How old are you?

3. Gender?

4. What is your highest level of education?

5. Have you taken any horse related training/education?

6. How do you respond to the statement below?

   The reason why I am a trotting trainer is that I am interested in animals and really like working with animals. (scale 1-10)

7. To what extent do you support yourself on your trotting horse business?
   (Scale 1-10: Hobby only - It's my only livelihood)

8. Do you have any employees (in addition to any family members)?

9. How would you like to describe your physical health?

10. How would you like to describe your mental health?

11. Do you enjoy training trotting horses?
    (Scale 1-10: No, certainly not - Yes, absolutely)

12. How many years have you worked with trotting horses?

13. Do you have any plans to stop training trotting horses?

14. Which type of license do you have (A or B)?

15. How many horses do you have in training?

16. Do you own all horses, or do you also train other people's horses?

17. Are you the only trainer at the premise or are there several other trainers on the premise/camp?

18. How do you keep your horses (loose housed, in boxes or tied-up in stalls)?

19. In addition to the horses being healthy and receiving good quality feed and water, what additional factors do you consider to be most important for your horses' welfare?
    Tick the boxes of the 3 factors you think are most important.

    That horses that are injured / fall ill quickly receive treatment

That the horses have plenty of space in the stable
That the horses are kept loose housed, i.e. not tied-up in stalls
That there is good air quality in the stable
That they get good care and management
That they feel safe with us who take care of them
That they are allowed to behave naturally
That they get daily access to outdoor runs/paddocks
That they have access to summer pasture
That they get physical activity / exercise
That they can socialize with other horses
That they get the opportunity for positive experiences, to feel positive emotions
That I comply with the animal welfare legislation
Other, namely (free text)

20. How would you describe the condition of your stable?

21. Below are a number of statements about animal welfare where you shall state how much you agree or disagree. Rate on a scale from 1 (do not agree at all) to 5 (fully agree):

    I feel that I would like to give higher priority to the horses' welfare.
    I sometimes have to down-prioritize the welfare of the horses to keep up with other activities.
    It is not possible to achieve a good financial situation in the business if I do not have good animal welfare.
    The fact that the horses are performing well is a clear sign of good animal welfare.
    I feel that the pressure to reduce costs is detrimental to the welfare of horses.
    I would have made several animal welfare improvements if it paid off financially.
    The increased attention from betters and the public to animal welfare is good.
    Good animal welfare is an important argument for the reputation of Swedish harness racing.
    Those who constantly violate animal welfare legislation destroys the confidence in Swedish harness racing.
    I myself have by name been negatively mentioned in media in connection to an animal welfare inspection.
    I have been negatively mentioned in the media in to an animal welfare inspection, but without my name or my stable being identified.

Part 2. Your experience on having inspections from different actors

22. How much do you agree or disagree regarding the following statements about getting animal welfare inspections from both the CAB and the STA? Rate the following statements on a scale from 1 (do not agree at all) to 5 (fully agree):

    I am well acquainted with the similarities and differences that exist in terms of requirements and assessments between the legislation and the standard of STA.
    I experience that the CAB and STA inspectors make about the same assessments, i.e. they usually assess my horses and my stables in the same way and get the same results.

I experience that STA makes tougher assessments than the CAB.
I believe that it is necessary with both governmental (CAB) and private (STA) inspections regarding animal welfare.
I find it easy to keep track of who comes and inspect my business and why they do it.
I would appreciate if there was better coordination between the various inspections.
I'm happy as it is.

23. How often do you think it is reasonable for a trotting trainer to receive animal welfare inspections (either from the CAB or STA)?

24. What do you think characterizes a good animal welfare inspector?
Tick the 3 most important characteristics for you.

The inspector is knowledgeable about the regulations.
The inspector is knowledgeable about horses and harness racing.
The inspector is knowledgeable about animal welfare.
The inspector is nice.
The inspector is knowledgeable in the administrative procedures and processing of cases/matters.
The inspector acts professionally.
The inspector makes uniform assessments, i.e. so that it will be the same for everyone.
The inspector is confident in his/her assessments.
The inspector shows understanding that I, as a trotting trainer, am under time and financial constraints.
The inspector shows understanding that minor deficiencies can always occur.
The inspector shows interest in my horses and my business.
The inspector is good at listening.
The inspector justifies and explain any non-compliances so that I understand why it is important to take action.
The inspector complies with the regulations.
The inspector is smooth and can make flexible assessments as long as the horses are well.
The inspector gives me advice on how I can live up to the regulations.
The inspector gives me advice about my horse husbandry and activities that extend beyond the regulations.
Other, namely [Free text]

25. Where do you turn to in the first place if you want to find out how the animal welfare inspections work (both the CAB and STA inspections)? Tick your 3 most important sources.

Other trotting trainers
Social media
My vet
My advisor
The inspector
Professional trade/horse journals
Meetings within the trotting business associations
The Swedish Board of Agriculture
The actor responsible for the inspection (i.e. CAB or STA)
STA (regardless inspection type)

Courses and conferences
Other [free text]

Part 3. Your expectations and experiences on animal welfare legislation and the official CAB inspections
Part 4. Your expectations and experiences on the Trotter Health Standard and STAs inspections.

<u>Author's comment</u>: Part 3 and 4 were separated in the questionnaire to the trainers, however, since they consist of almost exactly the same questions, just changing "CAB" with "STA" we have compiled these parts here in the English version. Hence, "regulation" below is both the legislation and the Trotter Health Standard.

26. State the extent to which you agree with the following statements. Grade the statements on a scale from 1 (do not agree at all) to 5 (fully agree):

    I follow the regulation because I want my horses to be well.
    I follow the regulation to avoid getting remarks from [CAB/STA].
    I follow the regulation because I believe that a serious entrepreneur must obey the law.
    I follow the legislation to avoid being deducted from EU subsides.
    I follow the Trotter Health standard to be able to keep my license.
    I follow the regulation because I and my business must have a good reputation in the area.
    I follow the regulation because society and the betters expect it.
    I follow the regulation because I want to contribute to a great deal of confidence in Swedish harness racing.
    I think it is easy to understand what is required of me in order for me to live up to the regulation.
    I am generally satisfied with the regulation.

27. Are there requirements in the regulation that you find complicated or difficult to comply with?

28. Are there rules in the regulation that you do not consider to benefit the welfare of horses in practice?

29. Are there requirements regarding administration and record keeping that you think are superfluous?

30. How much do you agree or disagree with the statements below regarding the [CAB/STA] inspections? Grade the statements on a scale from 1 (do not agree at all) to 5 (fully agree):

    [CAB/STA] inspections are needed to ensure that animal welfare is good with Swedish trotting trainers.
    I think it is good that the [CAB/STA] does (or may do) animal welfare inspections with me.
    I know how the [CAB/STA] chooses whom to inspect.
    I am well aware of the control guidelines that [CAB/STA] inspectors use when making their assessments.
    [CAB/STA] gives animal owners who are constantly non-complying far too many chances to take actions and make improvements.

[CAB/STA] is far too quick to take strict actions and sanctions when someone does not comply with the legislation.
I expect that an inspector from [CAB/STA] can give me substantial advice and guidance on how to live up to the regulation.
It is reasonable that anyone who violates EU legislation also risks a deduction on EU support.

31. Have you received any animal welfare inspection from CAB?

32. Have you received any animal welfare inspection from [CAB/STA] during the last 3 years?

    Answering 'yes' – these trainers were sent to Q37 about experiences
    Answering 'no' or 'cannot remember' - these trainers were sent to Q33 about expectations

33. Which statements below are in line with your **expectations** regarding the [CAB/STA] animal welfare inspections? Try to think about how it would feel if you knew that the [CAB/STA] will come tomorrow and do an animal welfare inspection? Grade the statements on a scale from 1 (do not agree at all) to 5 (fully agree):

    I know what is checked during the [CAB/STA] animal welfare inspection and why.
    I see the inspection as an opportunity to become updated on new rules and learn something new.
    The inspector's actions will correspond to what I expect from a good animal welfare inspector.
    The inspector will think that I have a good horse keeping.
    The inspector will find deficiencies in my horse keeping.
    The inspector will certainly have his/her "own agenda" in addition to checking the regulation.
    The inspector and I will agree on the assessments made by the inspector.
    Regardless of which individual inspector that comes from [CAB/STA], the control result with regard to my horse keeping will be the same.
    There will be discussions about how the regulation should be interpreted.
    The inspector will make tougher assessments if I start to question and discuss the assessments during the inspection.
    I think the [CAB/STA] inspector wants the best for me and my business.

34. Are you worried about the [CAB/STA] animal welfare inspection? (scale 1-10; Yes, really - No, not at all)

    Why do you feel the way you do? [Free text]

35. In what way would you prepare if you knew that you would soon receive an animal welfare inspection from [CAB/STA]?

    I do not need to prepare in any particular way.
    I look in old control reports.
    I am reading about the regulations.
    I am looking for information on what the inspection will focus on.
    I do last minute quick fixes on what I think will be included in the inspection to avoid remarks.
    Other, namely (free text)

36. Below are a number of statements about the purpose of the [CAB/STA] inspection, where you should state whether you agree or not. Grade the statements on a scale from 1 (do not agree at all) to 5 (fully agree):

The purpose of the inspection is to check if my business complies with the regulation.
The purpose of the inspection is to help me comply with the regulation.
The purpose of the inspection is to find non-compliances.
The purpose of the inspection is to give me advice so that I can have as good horse keeping as possible.
The purpose of the inspection is to make sure that my horses are well.
The purpose of the inspection is to control that I comply with the legislation so I can get my EU subsidies.
The purpose of the inspection is mainly to check upon other aspects, such as equipment, medication management and record keeping.

The questions that follow are for those that have had an [CAB/STA] inspection within the last 3 years – they answered about their ***experiences****.*

37. Think back to when you had your most recent animal welfare inspection from [CAB/STA]. On a scale from 1 (very negative) to 5 (very positive), how did you experience that inspection? (1 very negative, 2 negative, 3 neither or, 4 positive, 5 very positive)

38. How many inspectors from [CAB/STA] attended that inspection?

39. What age (in relation to yourself) and gender did the [CAB/STA] inspector/inspectors have?

40. Was the inspection announced in beforehand?

41. In what way did you prepare yourself before the inspection from [CAB/STA]?

I did not need to prepare in any particular way.
I looked in old control reports.
I read about the regulations.
I looked for information on what the inspection will focus on.
I did last minute quick fixes on what I think would be included in the inspection to avoid remarks.
Other, namely (free text)

42. Were you worried about the [CAB/STA] animal welfare inspection? (scale 1-10; Yes, really - No, not at all)

Why did you feel the way you did? [Free text]

43. Do you know what the reason was for the [CAB/STA]?

It was a planned routine inspection
It was an extra inspection, i.e. a follow-up due to previous non-compliances
Someone had reported to [CAB/STA] that I had deficiencies in my horse keeping

I do not know why I got an inspection.

44. The statements below relate to how you experienced the inspection you received from [CAB/STA]. Grade the statements on a scale from 1 (do not agree at all) to 5 (fully agree):

The inspection was fair.
The inspection was unnecessary.
The inspection disrupted my and the business's routines.
The inspection contributed to better horse keeping and better animal welfare.
The inspection took place under mutual understanding between the inspector and me.
It was easy to talk to the inspector, and I understood what the inspector was saying.
The inspector was nice and wanted something good for my business and me.
The inspector took my opinions and my skills into account.
The inspector was knowledgeable and acted professionally.
The inspector had the ability to explain and justify his/her assessments so that I understood.
The inspector and I agreed on the assessments.
The inspector appeared to be interested in my business.
The inspector seemed to think I had a good horse keeping.
The inspector seemed to have his/her "own agenda" in addition to checking for compliance.
When the inspector left, I felt well informed about what the inspection result was and what happens next.
The written inspection report was clear and easy to understand.
I myself behaved nicely and professionally during the inspection.

Is there anything else you want to let us know regarding how you experienced the inspection? (Free text)

45. Below are a number of statements about the purpose of the [CAB/STA] inspection, where you must state whether you agree or not. Grade the statements on a scale from 1 (do not agree at all) to 5 (fully agree):

I felt that the purpose of the inspection was to check if my business complied with the regulation.
I felt that the purpose of the inspection was to help me comply with the regulation.
I felt that the purpose of the inspection was to find non-compliances.
I felt that the purpose of the inspection was to give me advice so that I can have as good horse keeping as possible.
I felt that the purpose of the inspection was to make sure that my horses are well.
I felt that the purpose of the inspection was to control that I comply with the legislation so I can get my EU subsidies.
I felt that the purpose of the inspection was mainly to check upon other aspects, such as equipment, medication management and record keeping.

46. Did [CAB/STA] found any non-compliances at your horse premise?

Those trainers that did not have any non-compliances were sent to Q51.

47. What was the non-compliance about?

The management and welfare of the horses
The stables and interior design
Inadequate documentation/record keeping
Equipment
I do not remember
Other, namely (free text)

48. When you then read the inspection report, did new non-compliances emerge that the inspector had not pointed out during the inspection? That is, were you surprised when you read the inspection report?

49. The statements below relate to how you experienced the handling of non-compliances that were noted during the [CAB/STA] inspection. Grade the statements on a scale from 1 (do not agree at all) to 5 (fully agree):

The inspector explained/justified why something was a deficiency and how it risked affecting the horses' welfare.
I think the inspector made a correct assessment and management of non-compliances.
The inspector understood some minor deviations and did not list them as actual non-compliances.
Discussions arose about the non-compliances (or interpretation of the regulation).
I think the assessment became tougher because I questioned the inspector's assessment.
The inspector was confident in his/her assessment.
The inspector gave me advice on how I could rectify the non-compliance(s).
The inspector referred me to an adviser or other expert whom I could turn to for help and tips on how the non-compliance(s) could be corrected.
I got to be involved and influence how long time I had on me to reach compliance.
I know what happens if I do not take any actions to reach compliance.
Had another inspector from the [CAB/STA] made the inspection, the result would have been different.
It was difficult to rectify the non-compliance(s).
It was expensive to rectify the non-compliance(s).
I have rectified the non-compliance(s).

50. What did the inspection result in (when it came to actions from [CAB/STA]?

None
Oral advice
Remarks in an inspection report
Injunction [CAB]
Injunction with an administrative fine [CAB]
Fine [STA]
Deduction in EU subsidies [CAB]
I was forbidden to keep horses [CAB]
My horses were seized [CAB]
I was suspended from competing

Withdrawn license [STA]
Reported to the CAB [STA]
I do not remember

51. Did the inspector from [CAB/STA] mention that the control result could be different when [STA/CAB] performs an inspection?

52. Finally - is there anything else you would like to say about animal welfare inspections and regulations? [Free text]

Thank you for your participation!
