# Supplementary material for: Swedish Trotting Horse Trainers’ Perceptions of Animal Welfare Inspections from Public and Private Actors
Source: Animals (Basel). 2022 Jun 3;12(11):1441. doi: 10.3390/ani12111441 (PMC9179459; doi:10.3390/ani12111441)
Supplement: Supplementary file 1 [file animals-12-01441-s001.zip › animals-1693610-supplementary.pdf]

Table S1. Supplementary material on how responses to some of the categorical questions in the questionnaire were grouped into new groups before the statistical analysis.

Question	Original Response Categories	New Categories for Statistical Analysis
Geographical	Stockholm County<br>Uppsala County<br>Södermaland County<br>Östergötland County<br>Örebro County<br>Västmanland County	SE1
	Jönköping County<br>Kronoberg County<br>Kalmar County<br>Gotland County<br>Blekinge County<br>Skåne County<br>Halland County<br>Västra Götaland County	SE2
	Värmland County<br>Dalarna County<br>Gävleborg County<br>Västernorrland County<br>Jämtland County<br>Västerbotten County<br>Norrbotten County	SE3
Age	< 20 years<br>20-30 years	<= 30 years
	31-40 years	-
	41-50 years	-
	51-60 years	-
	61-70 years	-
	> 70 years	-
Gender	Woman	-
	Man	-
	Other/do not want to respond	*Removed, considered missing*
Education	Elementary/comprehensive school	-
	High school	-
	Vocational university	-
	College/university 1-3 years<br>College/university 4-5 years<br>College/university doctoral degree	College/university
Physical health	Very good<br>Good	Good
	Not very good<br>Bad	Bad
Mental health	Very good<br>Good	Good
	Not very good<br>Good	Bad
Work experience	1-4 years<br>5-9 years	1-9 years

	10-19 years	-
	20-29 years	-
	30-39 years	-
	40-49 years<br>> 50 years	> 40 years
Age/sex of inspector<br>*(multiple choice)*	One younger woman<br>Two younger women	*If only any of these alternatives were chosen:*<br>Only younger women/woman
	One younger man<br>Two younger men<br>One man in my age<br>Two men in my age<br>One woman in my age<br>Two women in my age<br>One older man<br>Two older men<br>One older woman<br>Two older women	*Any combination of these choices (also including younger women/women):*<br>Remaining ages and gender
	Don’t remember/cannot tell	*Removed, considered missing*
Was the inspection announced beforehand?	Yes, well in advance<br>Yes, some days before	Yes
	Only the same day<br>No	No, it was announced the same day or not at all
	I don’t remember	*Removed, considered missing*
